# Overexpression of a ‘*Paulownia fortunei*’ MYB Factor Gene, *PfMYB44*, Increases Salt and Drought Tolerance in *Arabidopsis thaliana*

**DOI:** 10.3390/plants13162264

**Published:** 2024-08-15

**Authors:** Guijie Luo, Weijia Cai, Hao Wang, Wei Liu, Xu Liu, Shizheng Shi, Lei Wang

**Affiliations:** 1 Suqian Institute of Agricultural Sciences, Jiangsu Academy of Agricultural Sciences, Suqian 223800, China; 2 Jiangsu Academy of Forestry, Nanjing 211153, China

**Keywords:** *Paulownia fortunei*, *PfMYB44*, salt stress, drought stress

## Abstract

*Paulownia fortunei (Seem.) Hemsl* is a *Paulownia* Sieb.et tree of the family Scrophulariaceae. It has become an important short-to-medium-term fast-growing multi-purpose tree species in China due to its rapid growth, strong adaptability, and excellent material properties. MYB transcription factors in plants have numerous and diverse functions, playing important roles in various aspects such as plant stress response. To investigate the function of MYB transcription factors in *Paulownia fortunei*, this study used PCR technology to clone the *PfMYB44* gene from *Paulownia fortunei*. The homology of PfMYB44 and SiMYB44 (*Sesamum indicum*) was the highest. Expression analysis results showed that *PfMYB44* was expressed in the root, stem, young leaf, and mature leaf of *Paulownia fortunei*, with the highest content in the root. Cold, drought, hot, salt, and ABA treatments could increase the expression level of *PfMYB44*. Overexpression-*PfMYB44* plants were constructed, and physiological and molecular analysis showed that *PfMYB44* could positively regulate salt and drought stresses. Under drought stress, the expression levels of *AtP5CS*, *AtCAT1*, *AtNCED3* and *AtSnRK2.4* in transgenic lines were significantly induced. Salt stress induced the expression of *AtNHX1*, *AtSOS1*, *AtSOS2* and *AtSOS3* genes, and the relative expression levels of these genes in transgenic *Arabidopsis* were higher. In conclusion, the functional study of *PfMYB44* laid a certain foundation for the study of *Paulownia* stress resistance, and was helpful to the study of its stress resistance mechanism and the cultivation of new stress resistance varieties.

## 1. Introduction

Drought, salinity, hot, and cold stresses can significantly alter the physiological and biochemical traits, gene expression patterns, and proteomic characteristics of plants, thereby having a significant impact on their growth, distribution, and yield formation. Drought seriously affects the growth and development of Paulownia, and can lead to various physiological and metabolic abnormalities of Paulownia plants, can even cause irreversible damage, eventually lead to the death of the whole plant, and seriously reduce the quality and yield of Paulownia. Under salt stress, paulownia is mainly affected by negative effects such as osmotic imbalance and ion toxicity [1,2]. Excessive Na^+^ affects the absorption and transportation of water and nutrients by root cells, leading to a large accumulation of Na^+^ in leaves and weakened photosynthesis, which in turn inhibits the growth and dry matter accumulation of paulownia [3]. Therefore, it is necessary to study the response mechanism of plants to abiotic stress in order to cultivate stress-resistant varieties.

Transcriptional regulation is one of the core links in plant resistance to adversity [4]. MYB transcription factors (TFs) are involved in plant growth and development, defense regulation, stress response, and play an active or negative regulatory role in these processes [5,6,7,8]. MYB TFs have a remarkable common feature: the N-terminal of its protein structure contains the MYB domain [9]. The MYB domain generally contains 1–4 incomplete repeats of the sequence R, each consisting of 50–53 amino acid residues, which determine the binding of transcription factors to different acting elements [10]. There are three α helices; the second and third helices form HTH structures with hydrophobic fragments, which help transcription factors bind to specific target genes, while the third helix is responsible for directly binding to the DNA [11]. According to the number of R-structures in the N-terminal, it can be divided into R1/R2-MYB, R2R3-MYB, R1R2R3-MYB and 4R-MYB subclasses [12]. In addition, the C-terminal contains a transcriptional activation domain that ensures transcriptional activity and participates in interactions between other transcription factors or DNA, resulting in diverse of regulatory roles for the MYB gene family [6].

In 1987, Pazares identified the first gene encoding the MYB transcription factor in plants, COLORED1 (C1), which encodes the MYB domain protein required for anthocyanin synthesis in maize [13]. Afterwards, researchers isolated MYB TFs from various plants such as *Arabidopsis*, *Chrysanthemum morifolium*, *Gossypium hirsutum*, tomato and soybean, and studied their protein functions [14,15,16,17,18]. *AtMYB44* actively responds to drought stress by regulating ABA signaling, stomatal movement, and root growth [14]. Overexpression of the *AtMYB12* gene significantly increases the accumulation of flavonoids, which can enhance the survival ability of plants under drought and oxidative stresses [19]. The *TaMYBsdu1* gene regulates wheat tolerance to salt and drought stresses [20]. Overexpression of *XsMYB44* and *BcMYB44* can resist drought stress by enhancing plant osmotic regulation ability and ROS homeostasis [21,22]. In addition, MYB21, MYB24, and MYB57 affect anther development and anther cracking in *Arabidopsis* by regulating the biosynthesis of flavonols or jasmonic acid, and similar functions and regulatory mechanisms are present in *Capsicum annuum* and *Nicotiana tabacum* [23,24,25,26,27].

*Paulownia fortunei* is an important high-quality fast-growing timber tree species, naturally distributed in many provinces of China, commonly found in low-altitude hills and plains [28]. It has a cultivation history of over 2000 years in China. *Paulownia fortunei* is drought-resistant and salt-resistant, playing an important role in wind and sand fixation, landscaping, and improving the ecological environment. Its wood is a good material for furniture manufacturing and musical instrument production [29]. Therefore, *Paulownia fortunei* has important ecological and economic value. The phenomenon of cold and salt is widespread and increasingly severe, which seriously restricts the economic production and sustainable operation of *Paulownia fortunei*. With the further study of transcription factor MYB44, it has been found that it responds to both drought and salt stress. However, there is currently relatively little research on the stress resistance of *Paulownia fortune*; the mechanism of its response to cold and salt stresses is especially still unclear. The stress resistance gene *PfMYB44* was isolated from *Paulownia fortunei*, and its expression changes were determined after different stresses, which laid a certain foundation for the study of the stress resistance of *Paulownia fortunei*, and was helpful to the study of the stress resistance mechanism and the cultivation of new stress resistance varieties.

## 2. Results

### 2.1. Isolation and Bioinformatics Analysis of PfMYB44

**Figure 1:**
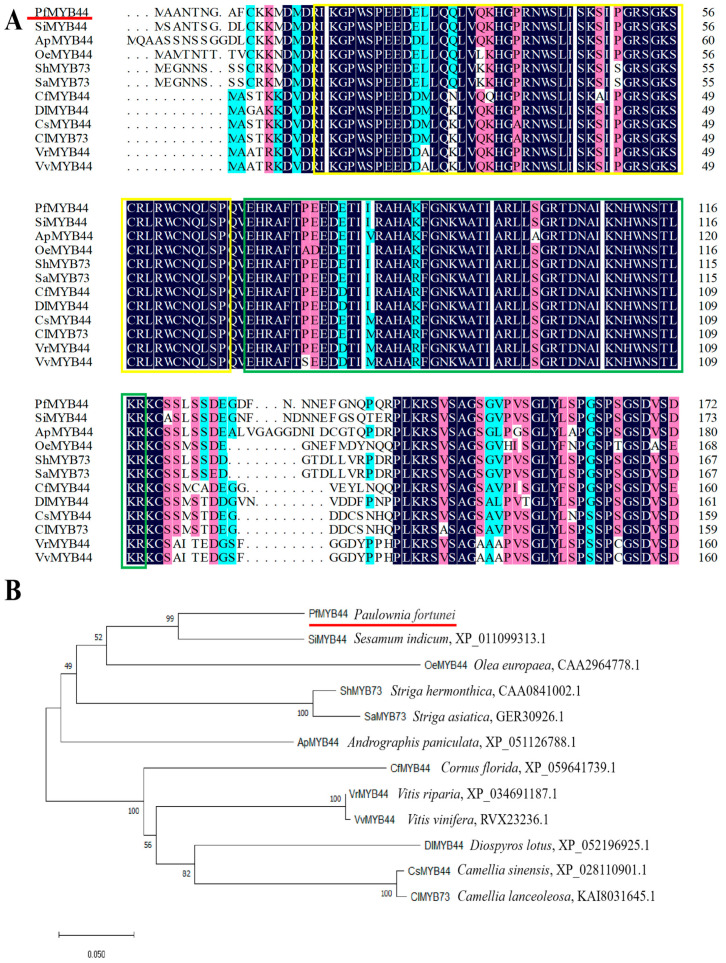
Homology analysis of PfMYB44. (**A**) Multiple-sequence alignment (**B**) and phylogenetic tree of PfMYB44 and other MYB protein species. The red line represents the target protein and the yellow and green boxes represent R-repeat structures.

The ORF (open reading frame) of the *PfMYB44* gene was obtained through PCR amplification, consisting of 1146 bp bases and encoding 381 aa (Figure S1). Domain analysis of *PfMYB44* revealed that it has two conserved SANT domains at the N-terminal, belonging to the R2R3 subfamily of MYB transcription factors. The first SANT domain contained 50 amino acid residues (positions 18–67), while the second SANT domain contained 49 amino acid residues (positions 70–118) (Figure S2B). The results of the secondary and tertiary structure of PfMYB44 predictions showed that the proportion of random coil was the highest at 63.25%, followed by the alpha helix (26.77%) (Figure S2A,C). In order to study the phylogenetic relationship between PfMYB44 and other species, Protein BLAST on the NCBI website was used to search for homologous genes of PfMYB44. A total of 11 homologous genes with higher alignment scores were selected for amino acid sequence alignment with PfMYB44, and a phylogenetic tree was constructed (Figure 1). PfMYB44 showed high conservation with homologous proteins in different species, and the closest relative species to PfMYB44 was *Sesamum indicum* (Figure 1B). It is speculated that *PfMYB44* may have a similar function to other MYB44 transcription factors, responding to both drought and salt stresses.

### 2.2. Subcellular Localization of PfMYB44 Protein

**Figure 2:**
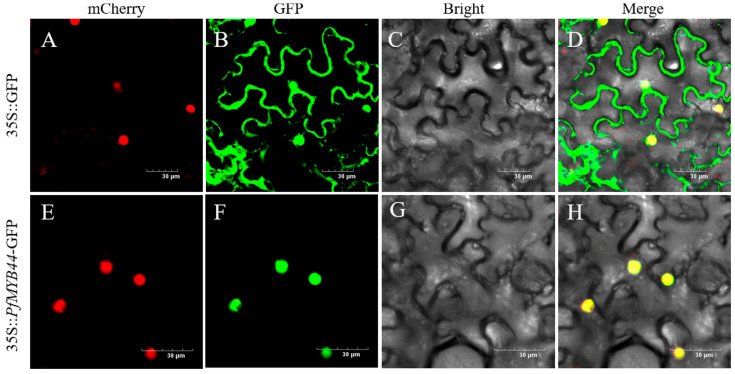
Subcellular localization of PfMYB44. (**A**,**E**) was mCherry, (**B**,**F**) was GFP, (**C**,**G**) was Bright, and (**D**,**H**) was Merge. Bar = 30 μm.

Using the online software BUSCA, PfMYB44 was predicted to be located in the nucleus. In order to further validate the prediction results, this study constructed PfMYB44 into pCAMBIA1300-GFP vector and transformed it into Agrobacterium GV3101. The permeate was resuspended and injected into tobacco leaves, and the subcellular localization of PfMYB44 was observed in tobacco epidermal cells to see if it coincided with the fluorescence of mCherry (Figure 2). *PfMYB44*-GFP fluorescence was observed at the site of the nucleus, while control GFP fluorescence was distributed throughout the cell. The subcellular localization results indicated that PfMYB44 was a protein that functions in the nucleus, consistent with the predicted results.

### 2.3. Expression of PfMYB44 in Paulownia fortunei

**Figure 3:**
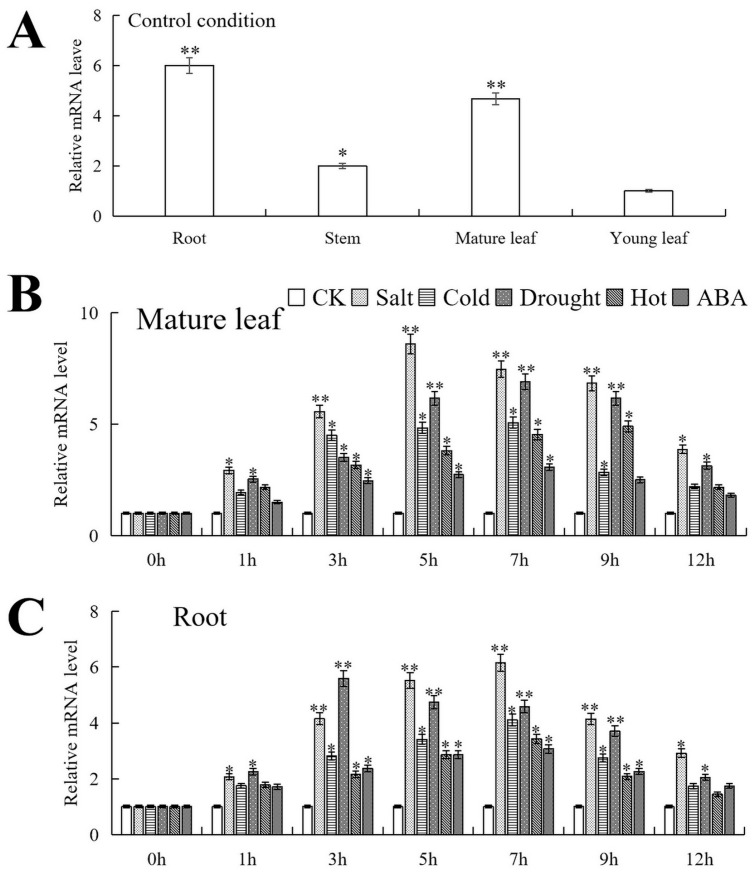
The expression of *PfMYB44* in *Paulownia fortunei*. (**A**) Expression levels of *PfMYB44* in different tissues of *Paulownia fortunei*. The expression level in young leaf was set to 1 for control. (**B**) Relative expression of *PfMYB44* under stresses in mature leaf and (**C**) root. The expression level of 0 h was set to 1 as the control. The SD represents error bar (*n* = 3). (*) *p* ≤ 0.05; (**) *p* ≤ 0.01.

*PfMYB44* gene was expressed in different tissues of *Paulownia fortunei*, but there were significant differences in expression patterns. Using the expression level of young leaf as a control, the *PfMYB44* gene had the highest expression level in root, approximately 6.0 times that of young leaf. Next were mature leaf and stem, which were 4.67 times and 2.0 times that of young leaf, respectively (Figure 3A).

Under salt, cold, drought, heat and ABA stresses, the expression of *PfMYB44* in mature leaf reached its peak at 5 h, 7 h, 7 h, 9 h and 7 h after treatment, and was 5.57, 5.07, 6.9, 4.9 and 3.07 times that of untreated leaf, respectively. In root, the expression of *PfMYB44* reached its peak value 3 h after drought treatment, while under salt, cold, hot and ABA treatments, the expression of *PfMYB44* reached its peak value at 7 h, which was 5.59, 6.16, 4.11, 3.43 and 3.07 times that of the control, respectively (Figure 3B,C). 

### 2.4. Heterologous Expression of PfMYB44 in Arabidopsis-Improved Salinity Tolerance

**Figure 4:**
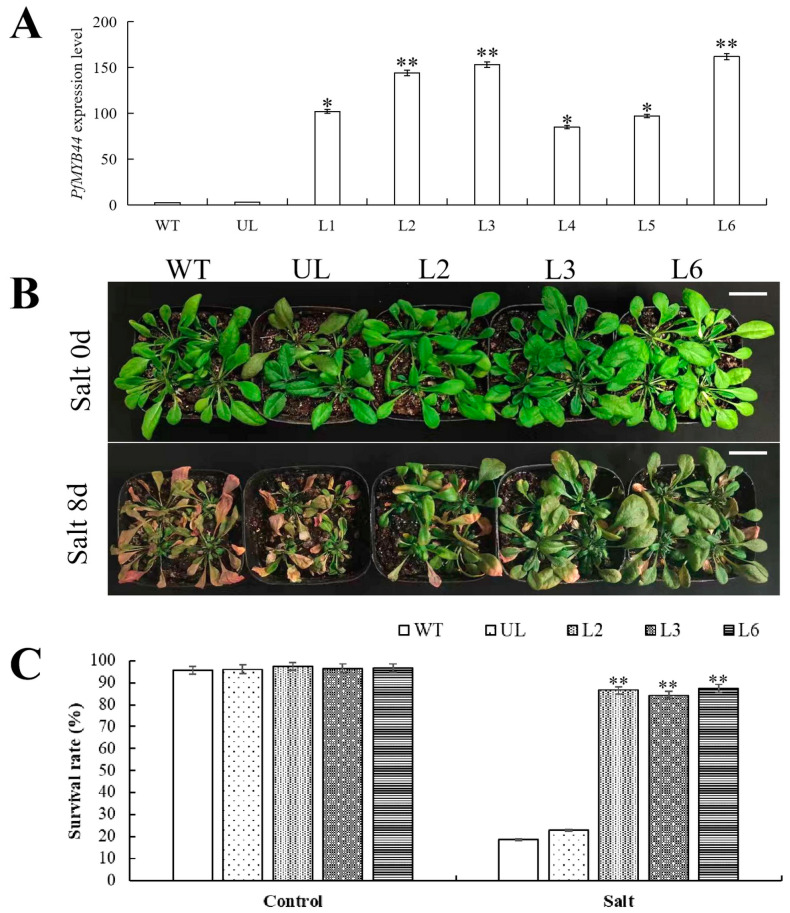
Overexpression-*PfMYB44* increased salt tolerance. (**A**) RNA level identification of transgenic plants. (**B**) Salt stress treatment for WT, UL and *PfMYB44* transgenic plants. Bar = 3 cm. (**C**) Survival rate statistical analysis of *Arabidopsis* under salt. The SD represents error bar (*n* = 3). (*) *p* ≤ 0.05; (**) *p* ≤ 0.01.

Overexpression of *PfMYB44* in *Arabidopsis* was achieved through the flower soaking method. The homozygous screening and identification of *Arabidopsis* were carried out by kanamycin, and a total of six initially screened positive *Arabidopsis* lines were obtained. RNA was further extracted from these six *Arabidopsis* lines; RT-qPCR detection showed that *PfMYB44* transgenic was successful, and the expression levels of L2, L3 and L6 were higher (Figure 4A).

According to the difference in expression levels of *PfMYB44* in transgenic *Arabidopsis*, three homozygous lines L2, L3 and L6 with higher expression levels were selected in this study. As shown in Figure 4B, under normal culture conditions (Salt 0 d), transgenic *Arabidopsis*, wild-type (WT) and unloaded line (UL) *Arabidopsis* plants grew well and in the same growth state, without significant phenotypic differences. Compared to the control group, the experimental group (Salt 8 d) showed wilting and yellowing in *Arabidopsis*. However, compared to the WT and UL *Arabidopsis* with severe wilting and yellowing, transgenic *Arabidopsis* showed a healthier growth state and a significantly higher survival rate (Figure 4C).

**Figure 5:**
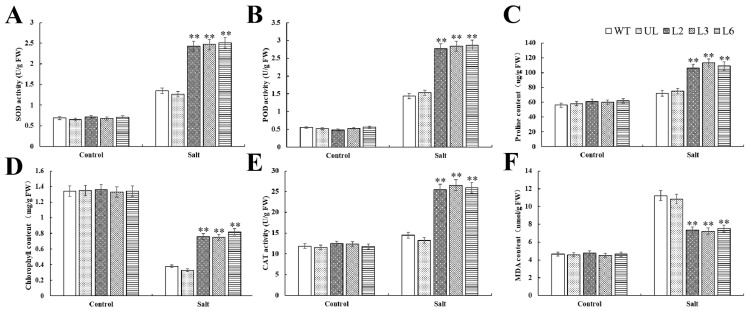
Statistical analysis of physiological indexes of plant stress resistance under salt stress. (**A**) SOD activity, (**B**) POD activity, (**C**) proline content, (**D**) chlorophyll content, (**E**) CAT activity, and (**F**) MDA content in *Arabidopsis* under salt stress. The SD represents error bar (*n* = 3). (**) *p* ≤ 0.01.

Salt stress breaks down the chlorophyll of plants, which in turn causes them to fail to photosynthesize and die. However, the chlorophyll of overexpression-*PfMYB44 Arabidopsis* was less damaged (Figure 5D). Salt stress increased the content of MDA and proline, and the activity of antioxidant enzymes in the experimental group. The transgenic line had higher proline content (Figure 5C). The accumulation of ROS is one of the important indexes to evaluate the harm of salt to plants. Therefore, the content of MDA and the activities of antioxidant enzymes in different lines under salt stress were determined. There was no difference between the control groups. MDA content in WT and UL of the experimental group was higher, indicating that they suffered from more severe osmotic stress (Figure 5F). In addition, the activities of catalase (CAT), superoxide dismutase (SOD), and peroxidase (POD), which are related to antioxidant activity, were lower, indicating that they accumulated more reactive oxygen species in their bodies (Figure 5A,B,E). These results indicated that *PfMYBYB44* can indeed enhance plant survival under salt stress.

### 2.5. The Expression of Salt Tolerance-Related Genes

**Figure 6:**
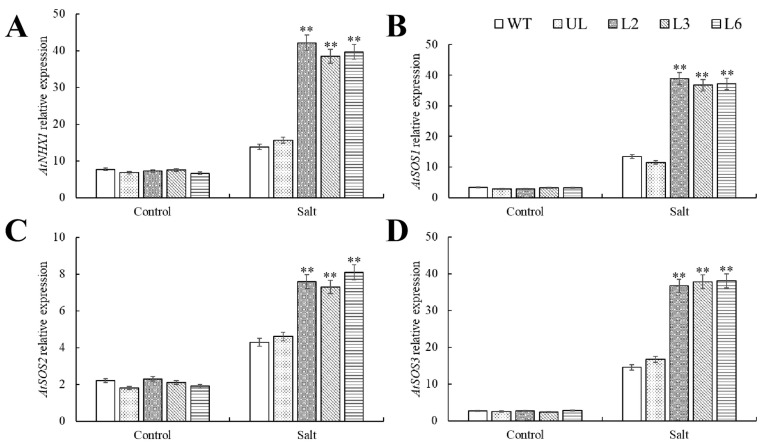
Expression of ion transport genes in WT, UL and transgenic plants under salt stress. The expression of (**A**) *AtNHX1*, (**B**) *AtSOS1*, (**C**) *AtSOS2*, and (**D**) *AtSOS3* under salt. The SD represents error bar (*n* = 3). (**) *p* ≤ 0.01.

In order to investigate the molecular mechanism of *PfMYBYB44* to improve salt resistance in plants, the expression of genes related to ion transport in transgenic *Arabidopsis* was determined. The relative expression levels of *AtNHX1*, *AtSOS1*, *AtSOS2*, and *AtSOS3* were not significantly different in the control group. However, salt stress induced their expression, and the relative expression of these genes in transgenic *Arabidopsis* was higher in the experimental group (Figure 6). It is suggested that *PfMYBYB44* may enhance plant salt tolerance by upregulating the expression of these genes related to ion transport.

### 2.6. Heterologous Expression of PfMYBYB44 in Arabidopsis-Improved Drought Tolerance

**Figure 7:**
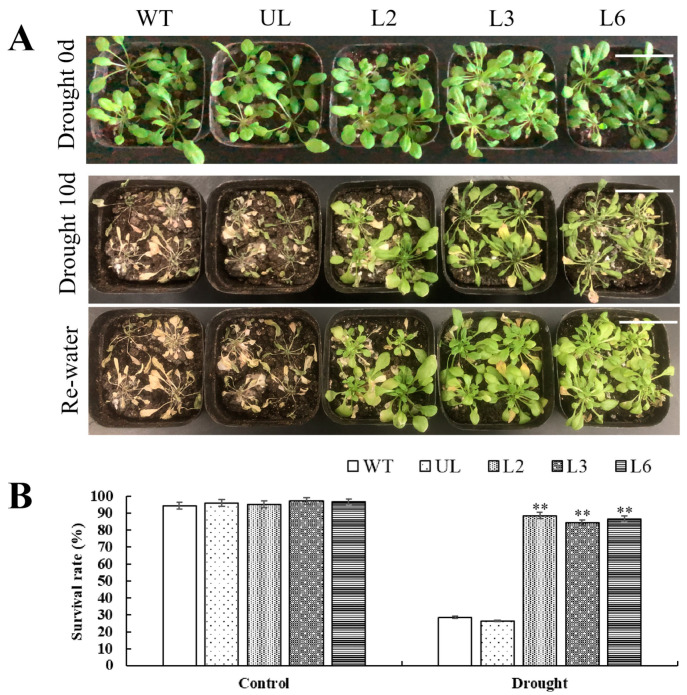
Overexpression-*PfMYB44* increased drought tolerance. (**A**) Drought stress treatment for WT, UL and *PfMYB44* transgenic plants. Bar = 3 cm. (**B**) Survival rate statistical analysis of *Arabidopsis* under drought. The SD represents error bar (*n* = 3). (**) *p* ≤ 0.01.

As shown in Figure 7A, all *Arabidopsis* lines cultivated under normal conditions (Drought 0 d) had good growth status and consistent growth, with no obvious phenotypic differences. Compared with the Drought 0 d, the WT, UL and transgenic *Arabidopsis* in the experimental group (Drought 10 d) showed obvious poor growth, and WT and UL *Arabidopsis* leaves showed more severe wilting phenomenon than transgenic *Arabidopsis*. After 3 d of rewater treatment, compared with WT and UL, the leaf status of transgenic *Arabidopsis* was obviously restored, and showed a higher survival rate (Figure 7B).

**Figure 8:**
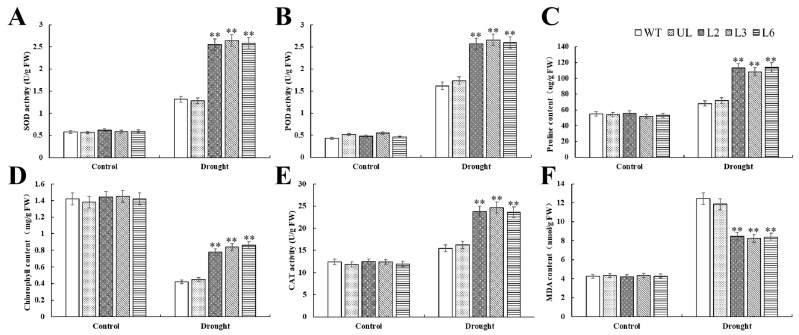
Statistical analysis of physiological indexes of plant stress resistance under drought stress. (**A**) SOD activity, (**B**) POD activity, (**C**) proline content, (**D**) chlorophyll content, (**E**) CAT activity, and (**F**) MDA content in *Arabidopsis* under drought stress. The SD represents error bar (*n* = 3). (**) *p* ≤ 0.01.

There were no significant differences in chlorophyll, MDA and proline contents among all *Arabidopsis* lines. MDA content of transgenic *Arabidopsis* in the experimental group was significantly lower than that of WT and UL, while the contents of proline and chlorophyll were opposite (Figure 8C,D,F). Transgenic *Arabidopsis* showed a greater increase in SOD, POD, and CAT activities under drought stress. This indicates that the overexpression of the *PfMYBYB44* gene in *Arabidopsis* can significantly reduce the accumulation of harmful substances in plants after drought stress, and improve the content of osmotic regulatory substances and antioxidant enzyme activity in leaves of *Arabidopsis* (Figure 8A,B,E).

### 2.7. The Expression of Drought Tolerance-Related Genes

**Figure 9:**
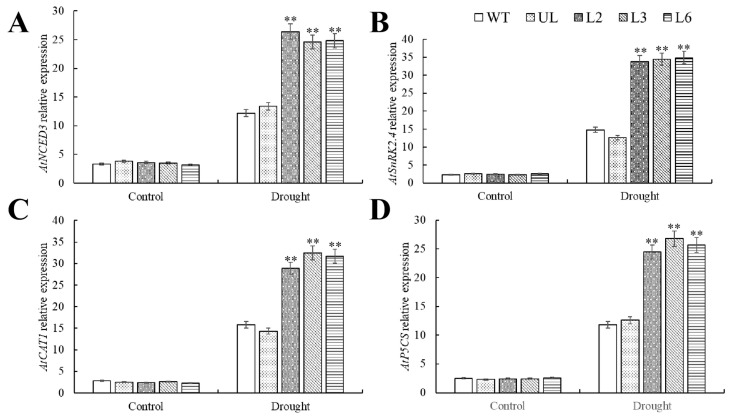
Expression of drought tolerance-related genes in WT, UL and transgenic plants under drought stress. Relative expression levels of (**A**) *AtNCED3*, (**B**) *AtSnRK2.4*, (**C**) *AtCAT1*, and (**D**) *AtP5CS* under drought stress. The SD represents error bar (*n* = 3). (**) *p* ≤ 0.01.

In order to investigate the molecular mechanism of *PfMYBYB44* to improve drought resistance in plants, we detected the expression of stress-related genes such as *AtP5CS*, *AtCAT1*, *AtNCED3*, and *AtSnRK2.4* using RT-qPCR. The expression levels of the above genes under normal treatment were similar in all lines. On the contrary, the expression levels of these genes in transgenic lines were significantly induced in the experimental group. Under drought stress, *PfMYB44* may improve plant survival by regulating the expression of these stress-responsive genes (Figure 9).

## 3. Discussion

The role of WRKY, bHLH, MYB and other gene families in regulating plant growth and development and responding to stress has been confirmed [30]. MYB transcription factors have highly conserved MYB domains, each of which generally contains three regularly spaced tryptophan residues. This special domain can form an HTH configuration, which binds to DNA to participate in regulation [31]. This experiment cloned the full length of the *PfMYBYB44* gene from *Paulownia fortunei* and conducted bioinformatics analysis on it. The PfMYBYB44 had two SANT domains, indicating that the PfMYBYB44 belonged to the R2R3-MYB transcription factor (Figure 1). This study found for the first time that MYB44 may play a positive role in regulating the response of *Paulownia fortunei* stress resistance, which lays a good foundation for later analysis of the stress resistance pathway of *Paulownia fortunei*.

Previous studies have shown that most MYB proteins are located in the nucleus, such as the GbMYBFL protein identified in *Ginkgo biloba*, which is located in the nucleus [32]. The ApMYBs protein in *Agapanthus praecox*, which is partially involved in the flavonoid biosynthesis pathway, is localized in the nucleus [33]. The results of subcellular localization in this study showed that PfMYBYB44 was located in the nucleus, which is consistent with the predicted results and previous studies (Figure 2).

Overexpression of *TaMYB31* can promote wax synthesis and expression of drought resistance genes, thereby improving drought resistance in *Arabidopsis* [34]. *AtMYB96* can respond to ABA, drought and salt treatments, and overexpression of *AtMYB96* positively regulates plant tolerance to drought and cold stresses through the ABA signaling pathway [35]. Besides serving as a positive regulatory factor, *OsMYB30* negatively regulates the cold tolerance of rice by reducing the content of maltose [36]. In this paper, the relative expression of *PfMYB44* was analyzed under salt, drought, hot, cold stresses and external ABA treatment. The results showed that the expression of the *PfMYB44* gene showed a trend of first increasing and then decreasing under these treatments (Figure 3B,C). The PfMYBYB44 gene can be expressed in root, stem, young leaf, and mature leaf, but there were certain differences in expression levels. The *PfMYBYB44* gene had the highest expression level in root, and its homologous gene *VhMYB44* also had the highest expression level in root [37]. It is speculated that *PfMYBYB44* and *VhMYB44* genes have similar functions and play the greatest role in roots (Figure 3A). This may be related to the physiological regulation of plants in response to stress, such as adjusting their water-use efficiency, maintaining cell stability, and maintaining energy metabolism balance.

Plants that are exposed to adverse environments for a long time will lose balance in their ROS metabolism system, leading to a rapid increase in intracellular free-radical content and peroxidation of unsaturated fatty acids, resulting in the destruction of the cell membrane structure. In addition, it will also cause the dynamic balance of reactive oxygen species production and clearance system to be disrupted, accelerating the process of plant cell death. Proline has hydrophilicity and plays an important role in stabilizing metabolic processes within plant cells and tissues. Under adverse conditions, the content of MDA and proline in plants reflects their stress resistance to a certain extent. The MYB TF participates in plant response to drought stress through multiple pathways [38]. ABA is one of the main hormones regulating drought stress, which can reduce water transpiration, decrease the leaf water loss rate, and improve plant drought resistance by controlling the opening and closing of stomata [39]. It was found that the expression of *SiMYB56* was upregulated by drought induction in millet, and overexpression of *SiMYB56* in rice regulated lignin synthesis and the ABA signaling pathway to enhance the drought resistance of transgenic rice [40]. In this study, we found that overexpression of *PfMYBYB44* significantly enhanced salt and drought resistance in *Arabidopsis* compared to the WT (Figure 4 and Figure 7). The determination of MDA content and free proline content showed that the degree of cell damage and plasma membrane peroxidation in transgenic lines was lower than that of the WT. This may be due to the stronger ability of transgenic *PfMYBYB44 Arabidopsis* to clear a large amount of harmful substances such as ROS produced by salt and drought stresses, effectively increasing the content of protective substances such as polyamines and peroxidase, making the plant more resistant to salt and drought stresses. Overexpression of *PfMYBYB44* significantly increases the activities of POD, SOD, and CAT in *Arabidopsis*, quickly clearing the accumulation of hydrogen peroxide and superoxide ions caused by drought in the body, thus improving the salt- and drought-resistant ability of plants (Figure 5 and Figure 8).

To cope with the disruption of ion dynamic balance caused by high salt environments, plants first choose to limit excessive Na^+^ entry and absorb more K^+^ to maintain intracellular homeostasis [41]. This process is usually handled by transport protein families such as SOS and HKT. SOS3 is usually located on the cell membrane and encodes Ca^2+^-binding proteins, responsible for receiving Ca^2+^ signals induced by salt stress [42]. SOS2 is located in the cytoplasm and encodes a serine/threonine protein kinase, which usually receives the salt stress signal transmitted by SOS3 and can form a transcriptional complex with SOS3 to further phosphorylate the downstream Na^+^/H^+^ transporter SOS1, thereby accelerating intracellular Na^+^ efflux and reducing the ion toxicity caused by salt stress [43]. NHX (Na^+^/H^+^ antiporter) is a cell membrane Na^+^/H^+^ antiporter protein that is located on the plasma membrane and vacuole membranes. It excretes Na^+^ from the cytoplasm or isolates it into vacuoles through reverse concentration gradient transport, reducing the ion toxicity of salt stress to cells [44,45]. The expression of ion transport-related genes *AtSOS1*, *AtSOS2*, *AtSOS3* and *AtNHX1* in different transgenic lines was detected, and the results showed that under salt stress, the expression of these genes in overexpression-*PfMYBYB44* lines was significantly upregulated compared with that of WT (Figure 6). It was further suggested that *PfMYBYB44* enhanced plant salt tolerance, which may be related to the enhancement of plant ion transport capacity. It is speculated that *PfMYBYB44* not only transports excessive Na^+^ to the extracellular space but also has a certain compartmentalization effect, causing plant vacuoles to receive certain salt ions to alleviate ion toxicity.

The expression pattern of NCED is closely related to the level of ABA in vivo. Rice *nced3* mutant is sensitive to salt and drought stresses, while overexpression of *OsNCED3* exhibits the opposite phenomenon [46]. *OsNCED4* and *OsNCED5* genes have also been confirmed to regulate the ABA level and stress resistance of rice under drought stress [47,48,49]. Catalase can directly act on H_2_O_2_, disproportionating it into H_2_O and O_2_, and protect cells from damage by ROS [50]. Proline is an amino acid synthesized in higher plants and plays a crucial role as a compatible osmotic substance under stress conditions. *P5CS*, as a key gene encoding proline synthesis, is significantly upregulated under osmotic stress induced by drought in species such as *Arabidopsis* and rice [51,52], and is highly responsive to osmotic stress. Protein kinase is an important core component of a plant stress defense mechanism, among which SnRK is a serine/threonine protein kinase, which plays an important role in plant stress response [53]. SnRK2 and SnRK3 are plant-specific protein kinases that actively participate in ABA signaling and various abiotic stresses in *Arabidopsis*, rice, wheat, and other plants [54,55]. In this study, *AtNCED3*, *AtP5CS*, *AtCAT1*, and *AtSnRK2.4*, four genes that have been shown to be mainly involved in drought stress, showed significantly higher expression levels in the overexpression of *Arabidopsis* after drought stress compared to WT (Figure 9). It can be inferred that under drought stress, *PfMYBYB44* is highly likely to affect the tolerance of *Paulownia fortunei* to drought stress by regulating the expression of key genes in these drought stress signaling pathways.

## 4. Materials and Methods

### 4.1. Sample Collection and Treatments

The *Paulownia fortunei* materials used in the experiment were cultured in the material culture room of the research group. The *Paulownia fortunei* seedlings (about 5–6 cm in height) with a good growth state and consistent growth were selected for stress treatment. Drought, salt, cold, hot and ABA treatment groups were set up, and the roots of the seedlings were immersed in Hoagland solution containing 15% PEG6000, 100 mM NaCl and 100 µM ABA for treatment, respectively. The seedlings cultured in Hoagland solutions were placed under a constant temperature and in humidity incubators at 4 °C and 37 °C, respectively [56]. The seedlings under normal conditions without any treatment were used as the control group. After material treatment for 0 h, 1 h, 3 h, 5 h, 7 h, 9 h, and 12 h, samples were taken from the leaf and root of seedlings in each group. After being frozen in liquid nitrogen, they were stored in a −80 °C ultra-low temperature refrigerator for extracting leaf RNA and conducting gene expression analysis under stress. The root, stem, young leaf and mature leaf of *Paulownia fortunei* seedlings were collected and cryogenically frozen with liquid nitrogen for gene tissue expression analysis.

### 4.2. Cloning and Bioinformatic Analysis of PfMYB44

The RNA of the above samples was extracted using a column extraction kit and reverse-transcribed to synthesize cDNA. The reagent kit was purchased from Vazyme (Nanjing, China) with item numbers RC411-01 and r323, respectively. Specific primers for the PfMYB44 gene were designed based on the transcriptome data of *Paulownia fortunei*, pPfMYB44-F (5′-ATGGCAGCAAATACTAATGGTG-3′) and pPfMYB44-R (5′-TCAATCAATCCTGCTGATTCC-3′). The complete *PfMYB44* gene fragment was obtained by PCR amplification using the cDNA. Purified PCR amplification products were transferred into T_5_ vector and transformed into Escherichia coli DH5α receptor cells. After screening the monoclonal bacterial solution for PCR identification, positive clones were selected and sent to the company for sequencing verification.

The amino acid sequence of PfMYB44 was submitted to the NCBI database, and homologous sequences of other species with high similarity were screened by Blastp comparison, and the phylogenetic tree was constructed by the NJ (neighbor-joining) method of MEGA 11. Multiple sequences of PfMYB44 and its homologous proteins were compared by DNAMAN 5.2. ExPASy ProtParam was used to predict the physicochemical properties of protein. SOPMA was used to analyze the protein secondary structure, SWISS-MODEL was used to predict the protein tertiary structure, and BUSCA was used to predict the subcellular localization of PfMYB44.

### 4.3. Expression Analysis of PfMYB44

This study used the cDNA extracted from each sample group in Section 4.2 as a template and performed RT-qPCR using the fluorescent quantitative dye reagent ChamQ Universal SYBR qPCR Master Mix (Vazyme, Nanjing, China). The specific primers for RT-qPCR of the *PfMYB44* gene were pPfMYB44-qF: 5′-CCACAGCGACCTTTGAAGA-3′ and pPfMYB44-qR: 5′-GACATGCGAAGCC GACAC-3′. Using *PfActin* as the internal reference gene, the specific primer sequence was pPfActin-F: 5′-AATGGAATCTGCTGGAAT-3′ and pPfActin-R: 5′-ACTGAGGACAATGTTACC-3′ [57]. The PCR program was set up according to the instructions of the reagent kit. The relative gene expression was calculated using the 2^−∆∆Ct^ method.

### 4.4. Vector Construction and Subcellular Localization of PfMYB44

Primers pPfMYB44-OE-F (5′-GAGCTCGGTACCGGGGATCCATGGGGAGACAATATATAAT GGTG-3′) and pPfMYB44-OE-R (5′-GCCCTTGCTCACCATGTCGACATCATCCTGCTGATTCA AT-3′) were used to add homologous arms to both ends of the PfMYB44 sequence. The overexpression-*PfMYB44* vector was constructed with pCAMBIA1300-GFP plasmid as the skeleton by the homologous recombination method and named 35S:: PfMYB44-GFP. The plasmid vector, which was successfully sequenced and validated, was transformed into Agrobacterium and positive clones were selected. The bacterial liquid was identified by PCR. 

Agrobacterium 35S::PfMYB44-GFP was injected into tobacco for nuclear localization and pCAMBIA1300-GFP Agrobacterium was used as blank control. The distribution and co-localization of GFP fluorescence and nuclear localization protein mCherry protein fluorescence in tobacco epidermal cells were observed by laser scanning confocal microscopy after 48 h of dark culture.

### 4.5. Generation of Transgenic Lines

The resuspension of Agrobacterium containing PfMYB44 was prepared and the dipping method was used to infect the flowering stage of *Arabidopsis* inflorescence. The seeds of T_1_ generation were harvested, dried in a 37 °C oven for one week, and placed in a cold environment of 4 °C for vernalization. After, they were disinfected with 75% anhydrous ethanol for 30 s and washed with sterile water three times, and disinfected with 10% NaClO solution for 3 m, washed with sterile water three times, and then seeded on 1/2 MS solid medium containing kanamycin (50 mg L^−1^). After 8–10 d, positive seedlings with robust roots and fresh green leaves were transplanted in the substrate (nutrient soil/vermiculite = 2:1). After 2 weeks of cultivation, T_1_-generation transgenic *Arabidopsis* were obtained. Leaf samples were collected, their RNA was extracted, and cDNA was obtained by reverse transcription as a template for RT-qPCR expression detection. The specific sequence primers were pPfMYB44-qF: 5′-CCACAGCGACCTTTGAAGA-3′ and pPfMYB44-qR: 5′-GACATGCGAAGCCGACAC-3′. The internal reference primers were pAtActin-F: 5′-CCCGCTATGTATGTCGC-3′ and pAtActin-R: 5′-AAGGTCAAGA CGGAGGAT-3′. Individual transgenic positive plants with high expression and subculture were harvested to obtain T_3_-generation homozygous plants for subsequent experiments.

### 4.6. Analysis of Related Physiological Indexes in Overexpression-PfMYB44 Arabidopsis

WT, UL and T_3_ transgenic *Arabidopsis* (L2, L3 and L6) seedlings with similar root length and growth on 1/2 MS solid medium were selected and transplanted into soil. Control, salt treatment and drought treatment groups were set up, respectively. After 3 weeks of normal growth of *Arabidopsis* seedlings in the soil, the control group maintained normal watering treatment. After the salt treatment group was watered with 300 mmol L^−1^ NaCl for 8 d, the phenotype and survival rate were observed. After 10 d of natural drought and 3 d of rewater treatment, phenotype and survival rate were observed, respectively. After phenotypic observation, the contents of MDA, proline and chlorophyll were determined, and the activities of antioxidant enzymes SOD, POD and CAT were determined. The kit instructions were followed for specific steps. The specific kit numbers are as follows: chlorophyll (Solarbio, Beijing, China: mlsw-E0781), MDA (Solarbio: BC0020), proline (Solarbio: BC0295), SOD (Solarbio:HP-A101F), POD (Solarbio:ZY7POD-1-Y) and CAT (Solarbio:YBSH150).

### 4.7. Expression Analysis of Stress-Related Genes in Overexpression-PfMYB44 Arabidopsis

Samples were taken from the leaves of *Arabidopsis* (WT, UL, L2, L3 and L6) before and after stresses; the RNA was extracted and reverse-transcribed into cDNA for analyzing the expression changes in stress-related genes in *Arabidopsis*. The primer sequences used were as follows: pAtP5CS-F/R: GATACGGATATGGCAAAGCG/CCAAGTCCAAATCGGAAACC; pAtCAT1-F/R: CGCCA TGCCGAAAAATACCC/CTTGCCTGTCTGAATCCCAGGAC; pAtNCED3-F/R: AGACAAATA CGCCGAAGA/CATACAGGACCCTATCACG; pAtSnRK2.4-F/R: GAGGAAATGGGGATGCAG AT/TTCTCACTTCTCCACTTGCG; pAtSOS2-F/R: GCAAGGGAAGAAGAAGAAGT/TCTCCG CTACATAACTGCC; pAtSOS3-F/R: GAATCCATCGCTCATCAA/CCATTTCTTCCTCTTCACA; pAtSOS1-F/R: TTCATCATCCTCACAATGGCTCTAA/CCCTCATCAAGCATCTCCCAGTA; pAtNHX1-F/R: AGCCTTCAGGGAACCACAAT/CTCCAAAGACGGGTCGCATG. The primers were from 5′ to 3′. Additionally, the *AtActin* was selected as the reference gene.

### 4.8. Statistical Analysis

All measurements were repeated three times. SPSS 26.0 software was used to conduct statistical scores of the data, and the ANOVA method was used to analyze the significant differences (* *p* ≤ 0.05, ** *p* ≤ 0.01).

## 5. Conclusions

This study preliminarily analyzed the bioinformatics characteristics and expression patterns of the *PfMYB44* gene in response to abiotic stress and ABA. It was found that *PfMYB44* belongs to R2R3-MYB and positively responds to drought, cold, hot, salt, and ABA stresses. Overexpression in *Arabidopsis* further confirms that *PfMYB44* is a positive regulatory factor for drought and salt stresses. Under salt stress, *PfMYBYB44* enhanced the ability of plants to transport Na^+^ and improve salt tolerance by upregulating the expression of ion transport-related genes *AtSOS1*, *AtSOS2*, *AtSOS3*, and *AtNHX1*. Under drought stress, *PfMYBYB44* was highly likely to affect paulownia’s tolerance to drought stress through the ABA pathway by regulating the expression of key genes in drought stress signaling pathways such as *AtNCED3*, *AtP5CS*, *AtCAT1*, and *AtSnRK2.4*. This provides an important reference for future exploration and exploration of such transcription factors, and further in-depth research is needed on their biological functions and molecular mechanisms in *Paulownia fortunei*.

## Data Availability

The original data for this present study are available from the corresponding authors.

## Supplementary Materials

The following supporting information can be downloaded at https://www.mdpi.com/article/10.3390/plants13162264/s1, Figure S1: Nucleotide and amino acid sequence of *PfMYB44*. The red underlined part was the R-repeated conserved domain; Figure S2: Structure analysis of PfMYB44 protein. (A) The secondary structure; (B) functional domain; and (C) tertiary structure of PfMYB44.
