# Supplementary figures and images for: Overexpression of a ‘Paulownia fortunei’ MYB Factor Gene, PfMYB44, Increases Salt and Drought Tolerance in Arabidopsis thaliana
Source: Plants (Basel). 2024 Aug 15;13(16):2264. doi: 10.3390/plants13162264 (PMC11360487; doi:10.3390/plants13162264)

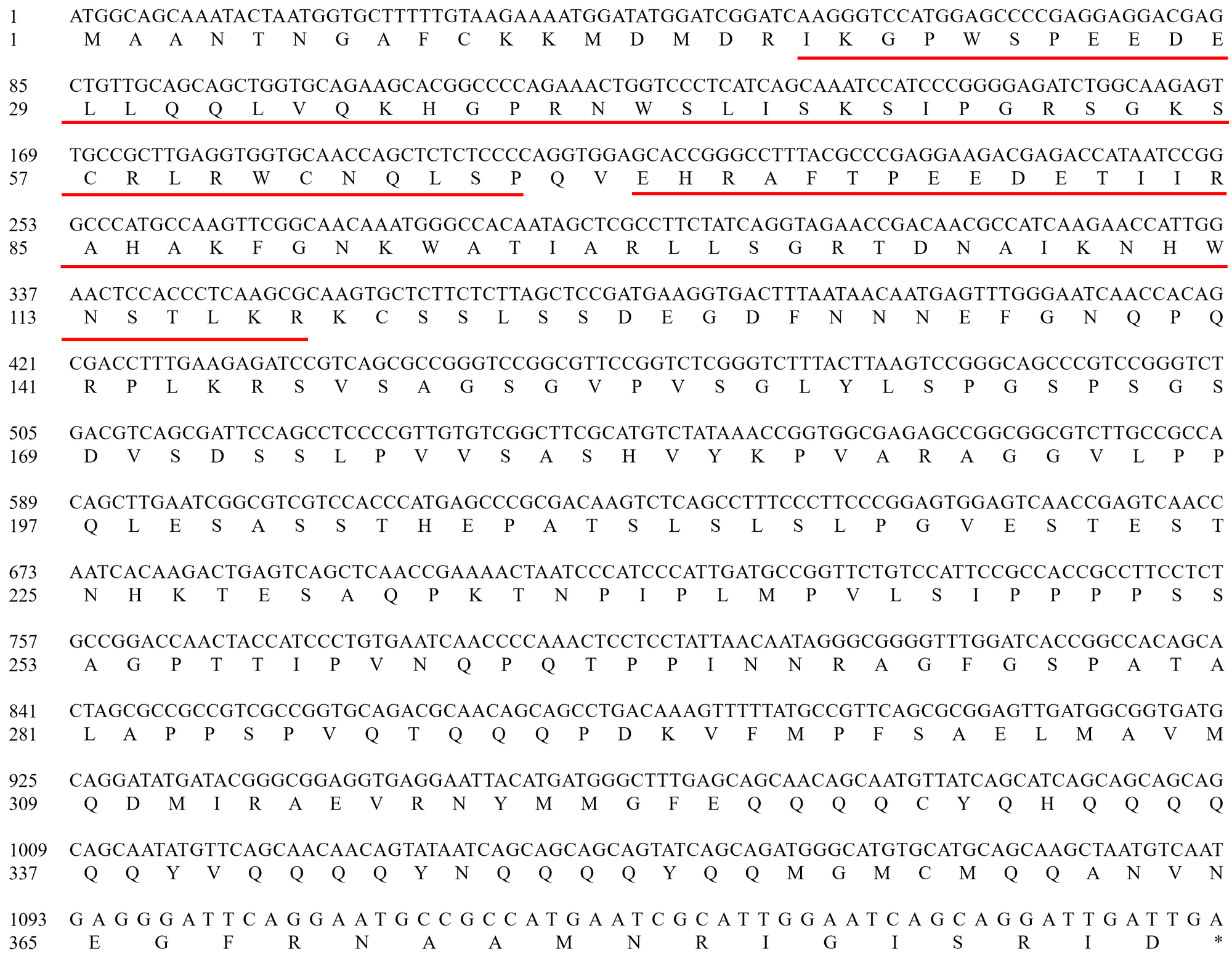

Supplement: Supplementary file 1 [file plants-13-02264-s001.zip › Figure S1.tif]

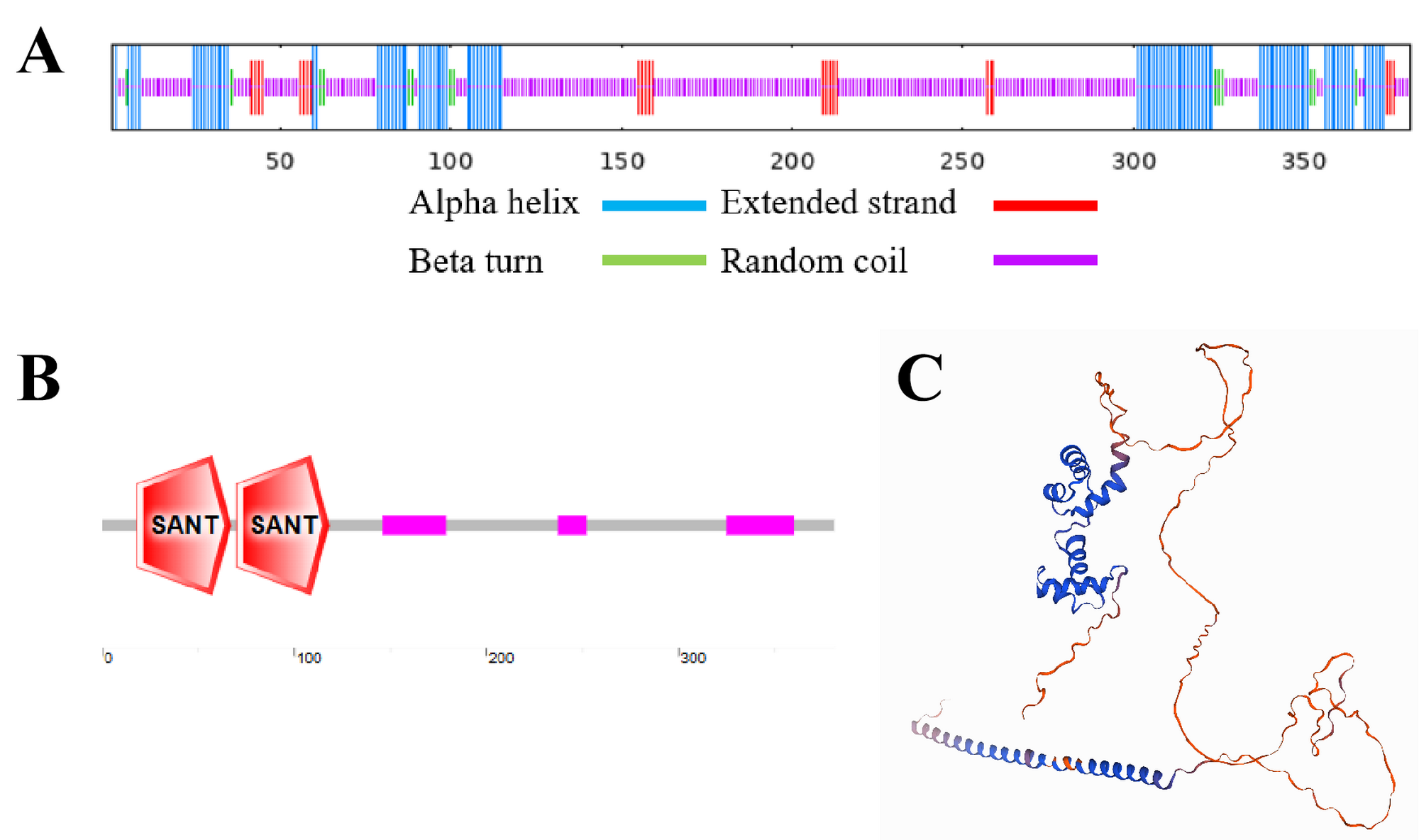

Supplement: Supplementary file 1 [file plants-13-02264-s001.zip › Figure S2.tif]
